# Understanding the Intersection Between Men’s Alcohol Use, Masculinity and Perpetration of Intimate Partner Violence: A Systematic Review and Meta-Analysis

**DOI:** 10.1177/15248380251383937

**Published:** 2025-11-12

**Authors:** Bree Willoughby, Koen Smit, Ingrid M. Wilson, Gail Gilchrist, Cassandra Hopkins, Amany Tanyos, Miro Saunders, Gedefaw Diress Alen, Anne-Marie Laslett

**Affiliations:** 1 La Trobe University, Melbourne, VIC, Australia; 2 Singapore Institute of Technology, Singapore; 3 King’s College London, United Kingdom; 4 Debre Markos University, Ethiopia

**Keywords:** alcohol, masculinity, male-to-female intimate partner violence, intersection

## Abstract

Male-to-female intimate partner violence (MFIPV) is a pervasive issue, with alcohol use and masculine norm endorsement identified as risk factors. However, research on how these two factors intersect with MFIPV perpetration is scarce. This review aims to understand if and how male alcohol use and masculinity intersect with MFIPV perpetration. A systematic search of Medline, PsycINFO, CINHAL, Global Health, and Web of Science was conducted. Papers underwent two levels of screening (title and abstract; full-text review) to determine eligibility, followed by methodological assessment and data extraction of all eligible studies. Three-level random effects meta-analyses were conducted to estimate pooled effect sizes for three outcomes of interest. Meta-regression analyses were performed to explore potential sources of heterogeneity across studies. Additionally, a narrative synthesis of qualitative data was undertaken. From 6,441 identified studies, 20 met the inclusion criteria. Meta-analysis results indicated greater alcohol use frequency (pooled odds ratio [OR] = 1.39, *p* = .004) and higher masculine norm endorsement (pooled OR = 1.12, *p* = .007) significantly increased the odds of MFIPV perpetration. Differences in sample size and measurement tools between studies accounted for significant proportions of observed heterogeneity. The interaction of alcohol use and masculinity on IPV was not significant (pooled OR = 1.00, *p* = .758). Further, the narrative synthesis provided insights into the conceptualization of masculinity and its intersection with alcohol. Alcohol use and masculinity independently predicted MFIPV perpetration; however, their interaction, while not statistically significant, remains underexplored. Future research should adopt a culturally grounded, intersectional approach to better understand their combined influence on MFIPV.

## Introduction

Male-to-female intimate partner violence (MFIPV) is globally endemic with over a quarter of ever-partnered women aged 15 to 49 estimated to have experienced physical and/or sexual intimate partner violence (IPV) at least once in their lifetime, with an estimated 13% of violence occurring within the past year (World Health Organization, 2021). The World Health Organization defines IPV as a “behavior by an intimate partner or ex-partner that causes physical, sexual or psychological harm, including physical aggression, sexual coercion, psychological abuse and controlling behaviors” (World Health Organization, 2021). A wealth of literature has identified risk factors for the perpetration of MFIPV (Clare et al., 2021; Schumacher et al., 2001). Among these, both endorsement of masculine norms and male alcohol use have consistently been shown to increase the risk of perpetration, with evidence also suggesting these factors also influence each other (Willott & Lyons, 2012). Despite this, much less is known about how these risk factors collectively interact to influence MFIPV. This review, therefore, aims to explore their intersection in relation to male-perpetrated IPV.

The link between alcohol use and MFIPV is well established. Research has found alcohol use increases the likelihood and severity of IPV globally (Graham et al., 2011; McKinney et al., 2010). Among men who do and do not drink, physical, sexual, financial, and emotional MFIPV perpetration significantly increased among those engaging in low risk drinking compared with abstainers (Shubina et al., 2023), with female partners of men who had been drinking twice as likely to report physical harm than if no alcohol was consumed (M. P. Thompson & Kingree, 2006). Analyses among alcohol dependent men have also shown consistently higher prevalence rates of MFIPV compared to men without an alcohol use disorder (Stanley, 2012). Qualitative research further strengthens the association between men’s alcohol use and MFIPV, with studies from the woman’s perspective identifying partner alcohol use as a prominent risk factor for victimization (Lennon et al., 2021; Wilson et al., 2024), describing MFIPV as a cycle of escalating violence which accompanies the partner’s progression through stages of intoxication and sobriety (Wilson et al., 2017). Analysis from the male perpetrator’s perspective has shown how alcohol consumption is used to justify the violence, void responsibility and claim that external factors such as relational stressors, are antecedent to drinking and subsequent violence (Cunha et al., 2024; Satyanarayana et al., 2015).

In addition to alcohol use, endorsement of masculine norms is another well-established risk factor for MFIPV. Masculinity has been conceptualized in a variety of ways across disciplines. Early research focused on behavioral and physical traits such as toughness, status and rejection of femininity (McCreary et al., 2005; E. H. Thompson Jr & Pleck, 1986). More recent research has taken a structural approach, most notably through R. W. Connell’s (1983) concept “hegemonic masculinity,”, which describes socially and culturally constructed attitudes and behaviors that establish male dominance and authority over women. This construct serves to perpetuate gender inequality and embody the most “ideal” way of being a man at a given time (R. W. Connell & Messerschmidt, 2005). However, masculinity is not a fixed universal construct, varying across countries and cultures (Costa Jr et al., 2001) and often viewed as a continuum ranging from “traditional” to “modern.” Multinational research by Williams and Best (1990) assessed sex role ideology from 30 countries comprising 14 cultures, finding those in traditional cultures (e.g., Nigeria, Pakistan) placed greater emphasis on patriarchal gender roles, where men held greater social and economic power, while those from egalitarian cultures (e.g., Finland, the Netherlands), endorsed less rigid role distinctions and greater gender equality. Given this diversity, this review includes a broad range of definitions to better understand its association with MFIPV.

Research on associations between masculinity and MFIPV has investigated how endorsement of masculine norms is linked to various forms of violence. Próspero (2008) identified that higher masculinity scores predicted higher psychological IPV perpetration, while Fleming et al. (2015) determined that men who held low gender-equitable attitudes and permissible attitudes toward violence against women were nearly twice as likely to perpetrate physical MFIPV. Another body of research has examined how failure to adhere to masculine norms, termed “masculine discrepancy stress,” is also a MFIPV risk factor. Higher stress has been associated with perpetrating severe physical, psychological and sexual MFIPV (Reidy et al., 2014; Sileo et al., 2022). Qualitatively, men have viewed violent behavior as within the normal confines of a masculine identity (James-Hawkins et al., 2019), noting violence as a strategy for disciplining a partner when disobedient as “part of being a man” (Sikweyiya et al., 2020). Further, Peralta and Tuttle (2013) identified a link between masculinity, the perceived need for relationship control and MFIPV, highlighting control over one’s partner and avoiding being controlled enables and maintains a sense of masculinity.

While male alcohol use and masculinity have both been linked to an increased likelihood of perpetrating IPV, evidence also suggests they influence one another (De Visser & Smith, 2007; Peralta et al., 2010). Historically, alcohol use has been viewed as a male-dominated pastime, with alcohol recognized as a marker and symbol of masculinity (Lemle & Mishkind, 1989; Willott & Lyons, 2012). De Visser and Smith (2007) found that men who drank more tended to be regarded as more masculine than men who did not. Further, public drunkenness and alcohol-related social behaviors were deemed masculine, with abstinence determined to detract from one’s perceived masculinity (De Visser & McDonnell, 2012). Male alcohol use has also been examined in relation to one’s level of endorsement of specific masculine norms. For example, Iwamoto et al. (2011, 2014) found that stronger levels of agreement with norms related to winning, risk-taking and playboy attitudes were positively associated with increased alcohol use among men. Moreover, greater risk of alcohol-related problems was linked to endorsement of norms emphasizing power over women, engagement in risk-taking behaviors and prioritizing work as central to one’s identity (Iwamoto et al., 2011).

Existing gender-transformative research, largely from Africa, has examined alcohol use, masculinity and MFIPV in the context of HIV. These studies often identify masculinity and alcohol misuse as key mechanisms for preventing and reducing violence and risky sexual behaviors. Hatcher et al. (2022) found that alcohol use, gender inequitable attitudes and MFIPV perpetration interacted to predict HIV risk among a sample of South African men. Similarly, Levtov et al. (2022) identified a pathway linking gender attitudes to violence through male alcohol use in Rwanda. Qualitative findings also suggested that changes in masculinity and alcohol use are synergistic in enhancing intervention efficacy (Hatcher et al., 2014). Further, a prior systematic review on gender equality and violence against women concluded that in the alcohol space, more nuanced, context-specific research is needed to examine how gender equality intersects with alcohol, particularly in relation to IPV (Roberts, 2011). Taken together, these findings highlight a consistent gap in research and interventions that address both masculinities and alcohol use as intersecting factors in MFIPV perpetration.

Despite the established link between male alcohol use and MFIPV, masculine norms and MFIPV, and alcohol use and masculinity, little is known about how these factors interact collectively. Therefore, this review explores the following question: How is men’s alcohol use associated with masculine norms and perpetration of MFIPV? Understanding the interplay between these two risk factors with MFIPV perpetration may inform the development of gendered health promotion approaches and targeted interventions that appropriately address the intersections of harmful masculinities, heavy alcohol use and partner violence.

## Methods

This systematic review and meta-analysis were conducted in accordance with the Preferred Reporting Items for Systematic Reviews and Meta-Analyses (PRISMA) guidelines (Page et al., 2021) and registered with the International Prospective Register of Systematic Reviews (PROSPERO; CRD42023462507).

### Databases and Search Strategy

Five major databases were searched for this systematic review and meta-analysis: Medline, PsycINFO, CINAHL, Global Health, and Web of Science. Additional hand searches were conducted in the following relevant key journals: *Men and Masculinities*, *Psychology of Men and Masculinities*, and *Sex Roles*. Further, reference lists from previous papers exploring the intersection between masculinity, alcohol use and IPV were scanned for additional studies that may not have been initially captured.

Three main search concepts were developed according to the aim of the review: alcohol use, masculinity, and IPV. Keywords pertaining to each concept were collated (see Supplemental Table 1) and entered into each database using Boolean operators. Medical subject heading terms were modeled after Medline subject headings, and adapted for available subject headings present within the other databases. Database searches were run from October 21 to 24, 2023. All identified studies were imported into Covidence (Veritas Health Innovation, n.d.) and underwent two levels of screening; title and abstract (Level 1) and full-text review (Level 2).

### Inclusion and Exclusion Criteria

Inclusion criteria were: (a) studies published in English from 2004 onwards, including peer-reviewed journals, theses and book chapters, (b) studies examining male-to-female partner violence perpetration, (c) studies with male participants aged 18 and older, (d) studies that assessed male alcohol use assessed by frequency and/or quantity questions, and (e) studies that measured masculinity via a scale (required only for quantitative studies), with results from the male perspective. Studies using the Gender Equitable Men (GEM) scale were also included. While this scale primarily measures gender-equitable attitudes, it also captures attitudes toward gender roles consistent with the broader conceptualization of masculinity applied in this review. There were no restrictions on study design (quantitative, qualitative or mixed-methods) or geographical location.

Studies were excluded if they: (a) were commentaries, reviews, editorials, unpublished work, case studies, intervention design (rather than outcome) or protocols, (b) focused on female-to-male partner violence perpetration, or same sex partner violence perpetration, or (c) focused only on female alcohol use.

All studies were screened and reviewed (full-text) independently by two authors. Any conflicts were resolved by a reviewer not involved in the initial assessment for that particular study. Study exclusion during the screening phase is documented in Figure 1.

**Figure 1.**
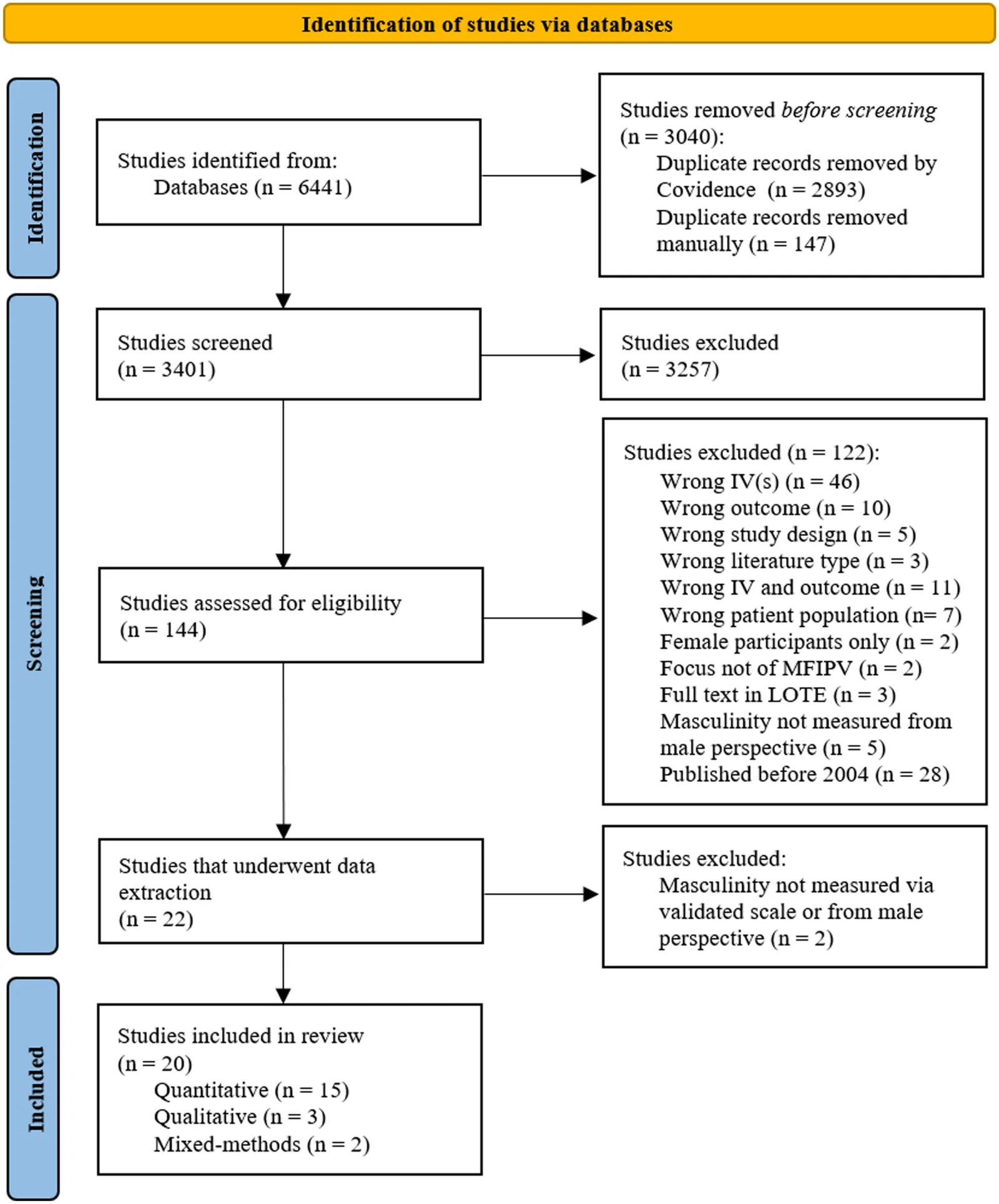
PRISMA flowchart of the systematic review search process. *Note.* PRISMA = Preferred Reporting Items for Systematic Reviews and Meta-Analyses.

### Data Extraction

The data were extracted using a template constructed in Covidence, which followed the PRISMA checklist. Extractions were completed by at least two reviewers independently, with conflicts resolved by discussion. The following domains were extracted for each exposure and outcome relationship (i.e., alcohol and IPV; masculinity and IPV; alcohol masculinity interaction and IPV) found within studies: (a) study characteristics (i.e., first author name, publication year, country of study, study period and study design), (b) sample characteristics (i.e., sample size, sex composition, mean age, sampling method), (c) data collection characteristics (i.e., study design, data collection method, tools for data collection, number of items in each tool, forms of IPV, IPV reference period), (d) type of exposure and outcome association assessed; and (e) findings (i.e., effect size, confidence intervals [CI], standard error, *p*-value). Following the extraction, two further studies were excluded due to not measuring masculinity from the male perspective or via a validated scale.

### Assessment of Risk of Bias

Study quality and risk of bias were undertaken independently by two authors per study, with conflicts resolved by a third author. The Mixed Methods Appraisal Tool (MMAT) was used, given its suitability for appraising reviews that include a mix of qualitative, quantitative and mixed-methods studies (Hong et al., 2018). The MMAT appraises studies across five categories of research design (qualitative, randomized controlled trials, non-randomized, quantitative descriptive, and mixed-methods), using five criteria per study type. These criteria assess sampling, measurement appropriateness and confounder control for non-randomized studies, adequate rationale, and integration of research components for mixed-methods studies, and methodological appropriateness, data quality, and overall coherence for qualitative studies.

### Data Analysis

A meta-analysis was conducted on the quantitative studies and a narrative synthesis was used for studies that included qualitative data. As all eligible studies employed a cross-sectional study design, odds ratios (OR) were used as the effect size. Studies with sufficient quantitative data, defined as those reporting an OR or providing enough information to calculate an OR, were included in the meta-analysis. For studies reporting different effect sizes, appropriate formulas were applied to generate OR approximations. To account for dependencies among multiple effect sizes nested within studies and differences across study methodology, three separate three-level random effects meta-analyses were conducted to estimate pooled ORs for each outcome. Due to no studies reporting on the association between “alcohol and masculinity,” this meta-analysis was not conducted.

Heterogeneity between studies was assessed using the *I*^2^ statistic, with values of 75% or greater defined as substantial heterogeneity (Deeks et al., 2023). Due to a substantial level of variance between studies, three-level random-effects meta-regression models were conducted to identify potential sources of heterogeneity. Both single and multivariable models were tested, incorporating study-level moderators such as sample size and measurement type. Potential publication bias was assessed through visual inspection of funnel plot asymmetry and statistically using Egger’s regression test. Statistical analyses were conducted using R software version 4.4.3 (R Core Team, 2025).

Several studies in this review utilized mixed-methods or qualitative methods, rendering them ineligible for inclusion in the meta-analysis. These data were narratively synthesized (Popay et al., 2006), and thematically explored masculinity, alcohol and MFIPV findings.

## Results

### Study Characteristics

A total of 6,441 studies were identified from databases searches, with 3,040 removed as duplicates. Following level 1 (3,401 studies) and level 2 (144 studies), screenings and the removal of two studies during data extraction, 20 studies were included in this review (Figure 1).

Characteristics of included studies are described in Table 1. All studies utilized a cross-sectional design with the majority employing quantitative methodology (15 studies). Just under half the studies (*n* = 8) were conducted in the USA, with the remainder conducted in low- and middle-income countries like South Africa (*n* = 4), Zimbabwe (*n* = 1) and Nepal (*n* = 1). In 8 out of the 20 studies, the mean age of participants was between 36 and 45 years. In seven studies, men were slightly younger (mean age ranged between 18 and 25 years).

**Table 1.** Summary of Study Characteristics.

Quantitative								
Author (Year)	Country	Study design	Study population	Measure(s) of alcohol use	Measure(s) of masculinity	Measure(s) of IPV	Type of IPV	Exposure(s) and outcome association(s) assessed
Abrahams et al. (2006)	South Africa	Cross-sectional	1,368 men aged 20 to 50+	Individual frequency and/or quantity questions	11 - item scale adapted from Rouse (1988)^a^	Individual items	Physical	Alcohol and IPV; masculinity and IPV
Cantor et al. (2021)	USA	Cross-sectional	350 student athletes (53% male, 45% female) aged 18 to 25	AUDIT	Ambivalent Sexism Inventory	Revised Conflict Tactics Scale	Any IPV	Alcohol and IPV; masculinity and IPV
Dasgupta et al. (2018)	India	Cross-sectional	1,081 heterosexual couples aged 18 to 30	Individual frequency and/or quantity questions	GEM scale	Measure from third wave of the National Family Healthy Survey	Physical and/or sexual	Alcohol and IPV; masculinity and IPV; alcohol, masculinity and IPV
Gilchrist et al. (2017)	Brazil and England	Cross-sectional	Ever-perpetrating IPV men in Brazil (*n* = 281) and England (*n* = 223) aged 19 to 73	AUDIT	Attitudes toward gender roles questionnaire	WHO multi-country study on women’s health and domestic violence	Any IPV	Alcohol and IPV; masculinity and IPV
Gottert et al. (2018)	South Africa	Cross-sectional	579 men aged 18 to 35	AUDIT	GEM scale; GRC/S scale	Adapted WHO questionnaire	Physical and/or sexual	Masculinity and IPV; alcohol and masculinity
Laslett et al. (2021)	Cambodia, China, Bangladesh, Indonesia, Papua New Guinea, Sri Lanka, Timor Leste	Cross-sectional	9,148 ever-partnered men aged 18 to 49	AUDIT	GEM scale	UNMCS on men and violence	Physical and/or sexual	Alcohol and IPV; masculinity and IPV; alcohol, masculinity and IPV
Lisco et al. (2015)	USA	Cross-sectional	329 heterosexual men aged 21 to 35	6 items from the NIAAA	Male Role Norms Scale; Masculine Gender Role Stress Scale	Revised Conflict Tactics Scale	Physical	Alcohol and IPV; masculinity and IPV; alcohol, masculinity and IPV
Lisco et al. (2012)	USA	Cross-sectional	205 heterosexual drinking men aged 21 to 35	6 items from the NIAAA	Ambivalent Sexism Inventory	Revised Conflict Tactics Scale	Sexual	Alcohol and IPV; masculinity and IPV; alcohol, masculinity and IPV
Lynch and Renzetti (2020)	USA	Cross-sectional	255 heterosexual men aged 18+ in a committed intimate relationship	3 items from the NIAAA	Ambivalent Sexism Scale	Severity of Violence Against Women Scale	Physical, psychological	Alcohol and IPV; masculinity and IPV; alcohol, masculinity and IPV
Machisa et al. (2016)	South Africa	Cross-sectional	416 adult men aged 18+	AUDIT	GEM scale	WHO multi-country study on women’s health and domestic violence	Physical and/or sexual	Alcohol and IPV; masculinity and IPV
Machisa and Shamu (2018)	Zimbabwe	Cross-sectional	2,838 men aged 18+	AUDIT	GEM scale	WHO multi-country study on women’s health and domestic violence; Sexual Relationship Power Scale	Any IPV	Alcohol and IPV; masculinity and IPV
Quantitative								
Author (Year)	Country	Study design	Study population	Measure(s) of alcohol use	Measure(s) of masculinity	Measure(s) of IPV	Type of IPV	Exposure(s) and outcome association(s) assessed
Miedema et al. (2022)	Republic of Kiribati	Cross-sectional	429 men who are currently partnered, aged 15 to 49	AUDIT	Gender Inequitable Attitudes Scale	WHO multi-country study on women’s health and domestic violence	Physical and/or sexual	Alcohol and IPV; masculinity and IPV
Pegram et al. (2018)	USA	Cross-sectional	556 single men aged 18 to 29	2 items from the NIAAA	Sexual Dominance Scale; Aggression Questionnaire (hostility subscale)	Sexual Experiences Survey	Sexual	Alcohol and IPV; masculinity and IPV
Renzetti et al. (2018)	USA	Cross-sectional	255 men aged 18+	3 items from the NIAAA	Ambivalent Sexism Scale	Severity of Violence Against Women Scale	Physical, psychological	Alcohol and IPV; masculinity and IPV; alcohol, masculinity and IPV
Warkentin (2008)	USA	Cross-sectional	514 male undergraduate students aged 18 to 27	Drink and Drug Habits Questionnaire	Gender Role Conflict Scale; Adversarial Heterosexual Beliefs Scale	Sexual Experiences Survey; Conflict Tactics Scale	Physical, sexual, verbal	Alcohol and IPV; masculinity and IPV
Qualitative								
Author (year)	Country	Study design	Study population	Description of alcohol Use	Description of masculinity	Description of IPV	Type of IPV	Outcome(s) assessed
Duff et al. (2020)	Namibia	Cross-sectional	10 males aged 25 to 45 convicted and sentenced for killing their intimate partner	Mentions of drinking alcohol to help manage anger, but made men less controllable	Mentions of feeling powerless in the relationship as a driver for men to act violently and regain power and control; strong sense of entitlement to access of sex	Mentions of stabbing, punching, slapping and fatal injuries to the head	Physical	Alcohol and IPV; masculinity and IPV
Puri et al. (2010)	Nepal	Cross-sectional	75 participants (39 married women; 36 married men) from separate households; 30 community leaders	Discussions of male alcohol use	Discussions of traditional cultural gender norms	Nature and experience of sexual violence; unwanted physical touch and forced sex from husbands	Sexual	Alcohol and IPV; masculinity and IPV
Wechsberg et al. (2013)	South Africa	Cross-sectional	67 participants aged 18 to 45	Information pertaining to community alcohol use and personal alcohol use at taverns/shebeens	Traditional gender roles	Information pertaining to partner violence	Physical, financial, sexual, coercive control	Alcohol and IPV; masculinity and IPV; alcohol and masculinity; alcohol, masculinity and IPV
Mixed-Methods								
Author (year) and methodology	Country	Study design	Study population	Measure(s) of alcohol use	Measure(s) of masculinity	Measure(s) of IPV	Type of IPV	Outcome(s) assessed
Peralta et al. (2010)	USA	Cross-sectional	11 heterosexual men convicted of IPV aged 25 to 55	Graduated-Frequency Measure; Drinker Inventory of Consequences	Questions regarding men’s beliefs about masculinity	Revised Conflict Tactics Scale	Physical, psychological, emotional, sexual, economic	Alcohol and IPV; masculinity and IPV; alcohol and masculinity; alcohol, masculinity and IPV
Tlapek (2016)	Rwanda	Cross-sectional	Survey interviews with 148 men aged 35 and over; semi-structured in-depth interviews with 29 married men and 16 of their wives	3 items from the University of Konstanz (based on DHS survey items)	WHO multi-country study on women’s health and domestic violence; DHS survey	Rwandan DHS survey (based on revised Conflict Tactics Scale); Baseline Survey on Sexual and Gender Based Violence in Rwanda (UNIFEM, 2008^b^)	Physical, sexual, emotional, financial	Alcohol and IPV; masculinity and IPV

*Note.* GEM = Gender Equitable Men; UNMCS = United Nations Multi-Country Study; NIAAA = National Institute of Alcohol Abuse and Alcoholism; GRC/S = Gender Role Conflict/Stress; IPV = intimate partner violence; WHO = World Health Organization; AUDIT = Alcohol Use Disorders Identification Test.

a Rouse (1988).

b UNIFEM (2008).

The eligible studies used a variety of measurement tools assess alcohol use, the most common being the Alcohol Use Disorders Identification Test (AUDIT; Babor et al., 2001), followed by the National Institute of Alcohol Abuse and Alcoholism (NIAAA) recommended consumption questions (National Institute on Alcohol Abuse and Alcoholism, n.d.). A variety of standardized tools were used to measure masculinity and IPV, with a handful of studies employing individual measures (e.g., IPV questions from a national health survey, masculinity items adapted from existing scales). However, the GEM scale (Pulerwitz & Barker, 2008) and hostile subscale of the Ambivalent Sexism Inventory (Glick & Fiske, 1996) were the most commonly used tools for assessing masculinity, and the WHO Multi-Country Study on Women’s Health and Domestic Violence (García-Moreno et al., 2005) and the Revised Conflict Tactics Scale (Straus et al., 1996) were the most common IPV tools.

The types of IPV and the reference period assessed ranged across studies, with the majority analyzing physical and physical/sexual perpetration that occurred in the past 12 months. Regarding outcomes, 19 out of 20 studies analyzed the association between alcohol and MFIPV, with all reporting on the association between masculinity and MFIPV perpetration. Only three studies explored the relationship between masculinity and alcohol, with under half examining (8 out of 20) the intersection of alcohol and masculinity on MFIPV perpetration.

The majority of included studies were of high quality (90%) as according to the MMAT (see Supplemental Table 2), reflecting appropriate study design and clear data collection methods and reporting. Although some studies were rated as moderate to low quality, all were included due to their conceptual relevance to the aims of this review.

### The Relationship Between Alcohol Use and MFIPV

Of the 17 studies that included quantitative findings concerning alcohol and MFIPV, 14 specifically reported relative effect sizes (mainly ORs) assessing this relationship. A meta-analysis of these studies, comprising a total of 17,840 participants, found that more frequent and/or heavier alcohol use was significantly associated with increased odds of reporting MFIPV perpetration (pooled OR = 1.39; 95% CI [1.14, 1.69], *p* = .004), with substantial total heterogeneity (*I*^2^ = 99.5%; Figure 2). The majority of this variance was observed between studies rather than within (98.1%; τ² = .1093 and 1.9%; τ² = .0021, respectively).

**Figure 2.**
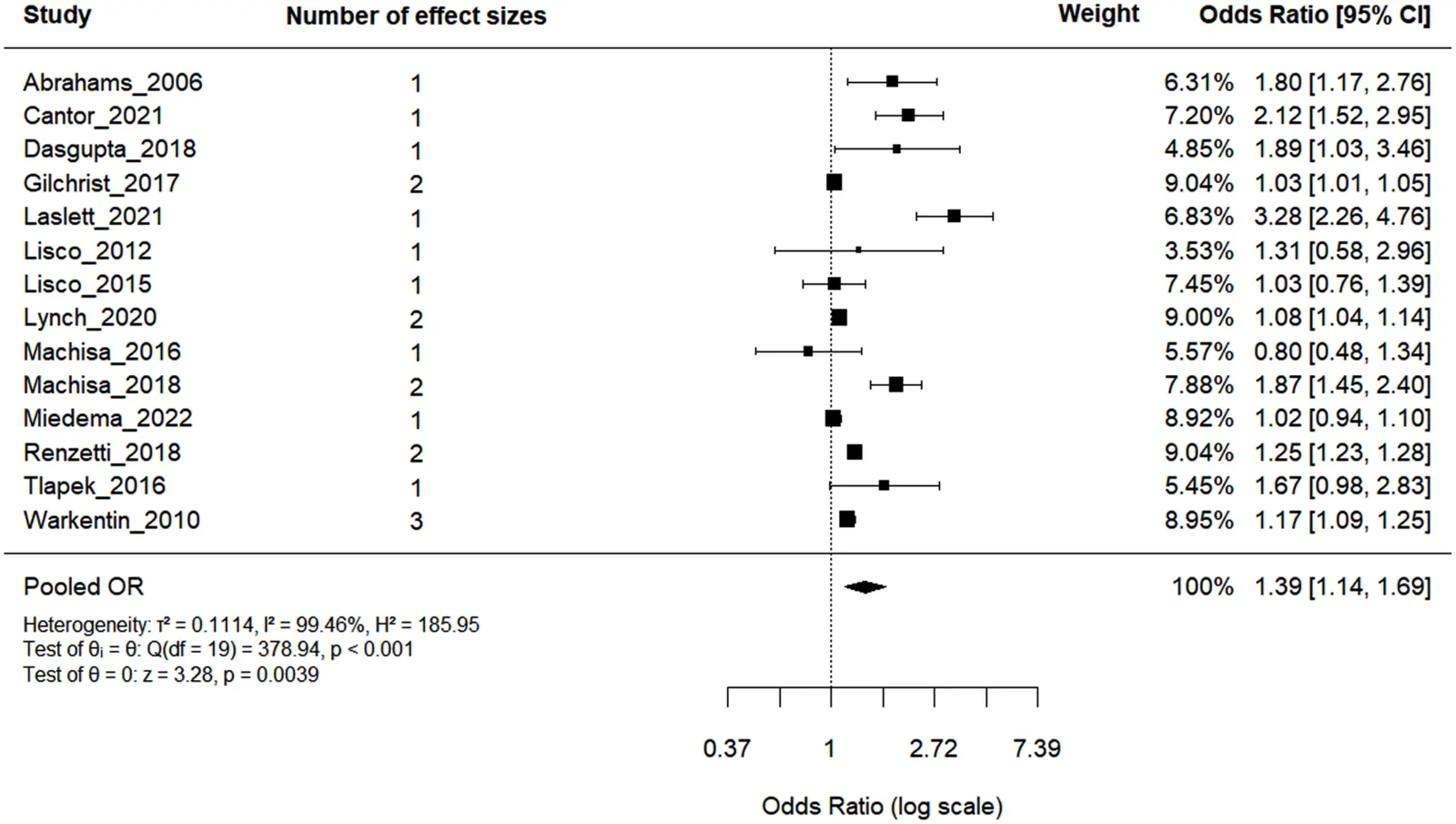
Forest plot of the association between alcohol use and MFIPV.

Given the substantial heterogeneity observed, meta-regressions were conducted to examine potential moderating factors. In single moderator models, only the sample size of the study significantly explained variation between effect sizes. Despite this, additional variables (e.g., measurement tools, type of IPV, IPV reference period, country of study and country income status) were analyzed together to assess potential combined influence. Collectively, sample size and IPV and alcohol measurement tools significantly explained some of the variance (QM[*df* = 9] = 45.46, *p* < .001), 87.7% total heterogeneity and 89.2% of between-study differences. Specifically, compared to large sample sizes (1,000+), small (< 300; OR = 0.59, *p* < .001) and medium (300–999; OR = 0.56, *p* = .003) studies had significantly weaker associations. Studies that measured alcohol use via the NIAAA or individual frequency questions reported significantly lower effect sizes compared to the AUDIT. Interestingly, studies using the WHO Multi-Country IPV measure showed significantly weaker effects than individual/other IPV measures. However, a significant proportion of residual heterogeneity remained (QE[*df* = 10] = 77.38, *p* < .001), suggesting additional unmeasured sources of variation.

### The Association Between Masculinity and MFIPV

Quantitative assessment of the association between masculinity and IPV was reported in 15 studies, with a total of 21 individual effect sizes (mainly ORs). The meta-analysis of these studies (Figure 3) revealed that greater endorsement of masculine norms was significantly associated with greater odds of reporting MFIPV perpetration (pooled OR = 1.12; 95% CI [1.03, 1.21], *p* = .007). Again, substantial total heterogeneity was observed (*I*^2^ = 98.37%), with 98.2% attributed to between-study variance and 1.8% to within-study.

**Figure 3.**
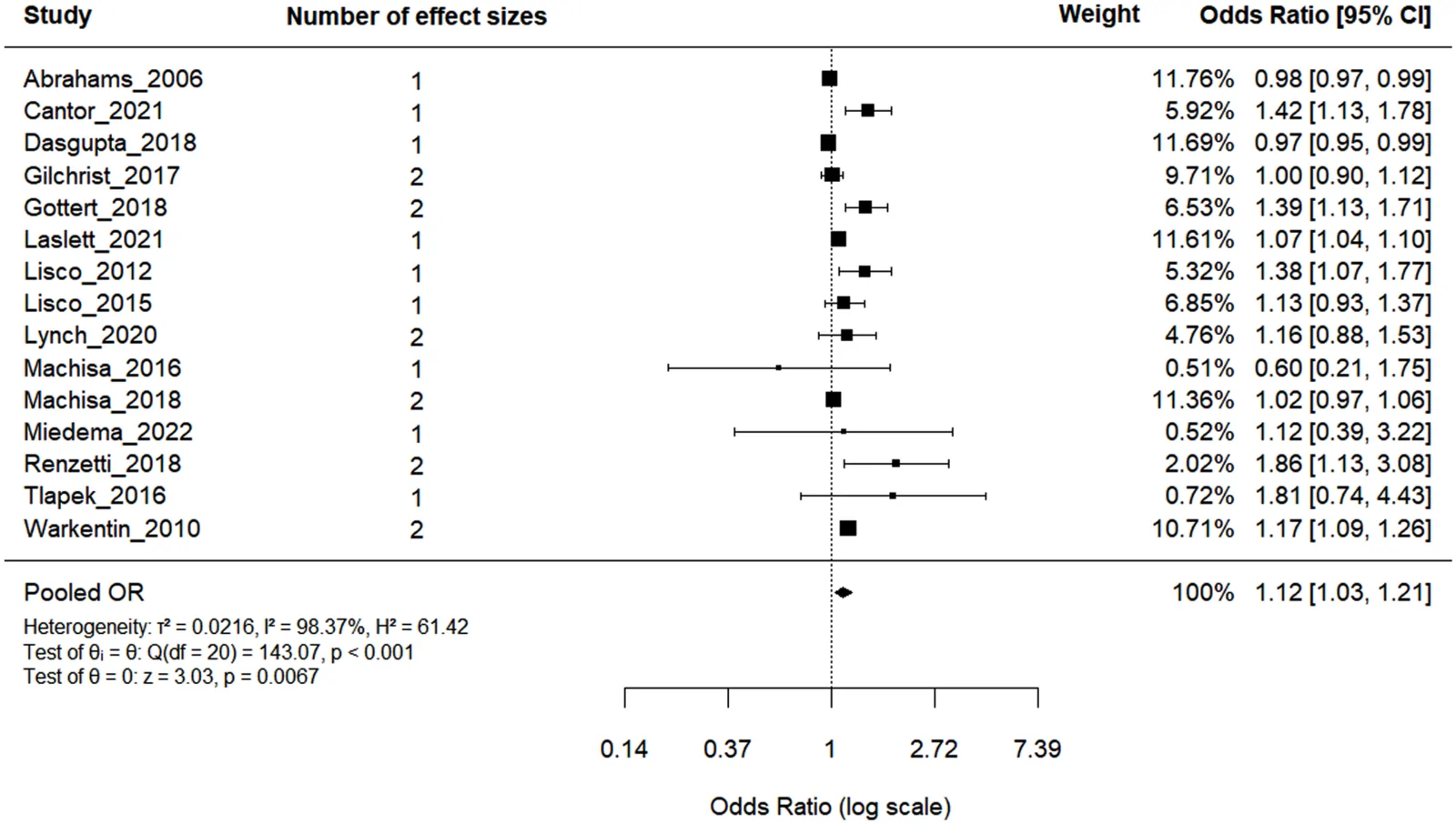
Forest plot of the association between masculinity and MFIPV.

Single moderator models revealed that sample size, country, IPV reference period, and masculinity and IPV measures each significantly explained variation in effect sizes. Multiple combinations of these variables were run together in multivariable models, with a model of sample size and masculinity tool emerging the best fit and significantly explaining 88% of the total heterogeneity (QM[*df* = 5] = 30.0614, *p* < .0001), and 90.6% of between-study differences. Specifically, medium sample sizes (300–99) reported significantly weaker associations compared to larger studies (OR = 0.56, *p* = .001) and studies measuring masculinity via the Ambivalent Sexism Inventory had significantly stronger effects than those using individual, less known masculinity tools (OR = 1.35, *p* = .005). Nonetheless, significant residual heterogeneity persistent (QE[*df* = 15] = 59.39, *p* < .001), indicating the presence of unexplored sources of variance.

### Alcohol, Masculinity and MFIPV

Six studies specifically examined and reported the quantitative association between the interaction of alcohol use and masculinity on MFIPV (see Figure 4). While individual studies show varying directions and strengths of associations, the overall pooled association suggests no significant relationship (pooled OR = 1.00; 95% CI [0.91, 1.09], *p* = .758). A high proportion of heterogeneity was found (*I*^2^ = 75.85%), with 100% of the variation between effect sizes arising from differences within studies rather than between studies.

**Figure 4.**
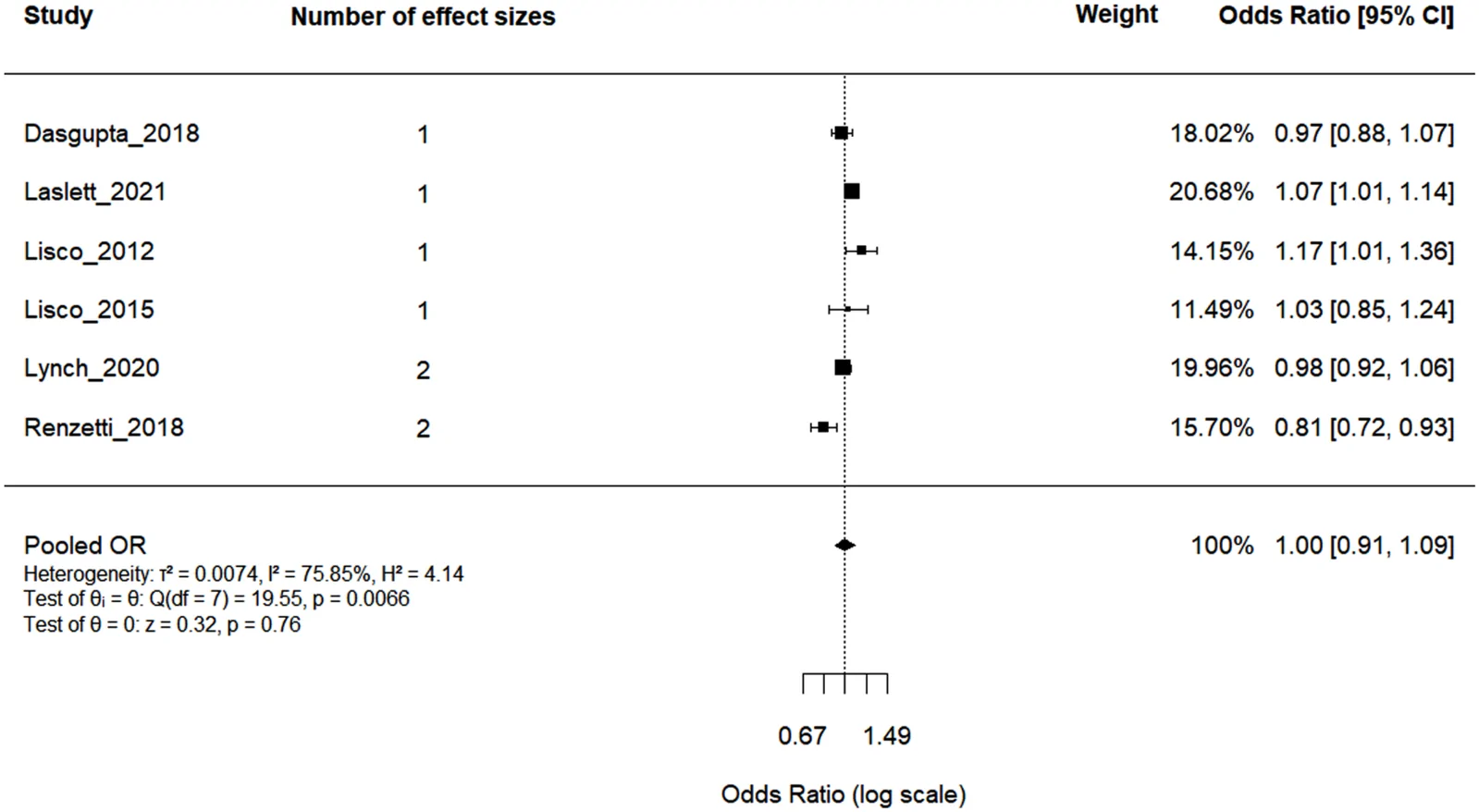
Forest plot of the association between the interaction of alcohol use and masculinity on MFIPV.

Given the small number of studies (*k* = 6) and effect sizes (*n* = 8), meta-regression models testing potential moderators of within-study heterogeneity were statistically underpowered. Although exploratory single moderator models were conducted, none of the moderators examined were significant. Thus, no clear sources of heterogeneity were able to be identified.

### Narrative Synthesis

Several studies in this review utilized mixed-methods (*n* = 2) or qualitative methods (*n* = 3) of data collection and analysis, rendering them ineligible for inclusion in the meta-analysis. Therefore, a narrative synthesis of qualitative data was conducted, identifying three key themes across the studies

#### Masculinity as a Framework for Dominance and Control

Discussions surrounding masculinity were consistently linked to justifications of control and violence, particularly when one’s authority was challenged or questioned. This association was evident across diverse settings and populations including incarcerated male perpetrators in Namibia (Duff et al., 2020), economically disadvantaged men in the USA (Peralta et al., 2010) and husbands of young married women in Nepal (Puri et al., 2010). For example, in Tlapek (2016) married men described giving orders without question as an essential part of masculinity, stating “I believed a man has to give orders” and suggesting female partners who disagree should suffer consequences (i.e., physical abuse). Perceived deficiencies in masculinity, such as lack of stable employment were linked to MFIPV, with men “prov[ing] masculinity” through abuse of intimate partners in order to regain a sense of control (Peralta et al., 2010). Attitudes concerning sexual entitlement were also common, with many defining manhood in terms of sexual activity frequency (Puri et al., 2010), subsequently viewing cheating as normal and acceptable (Duff et al., 2020). Specifically, Wechsberg et al. (2013) noted women’s lack of sexual autonomy due to this entitlement, with refusal of sex in intimate relationships leading to threats of cheating or sexual violence.

#### Interaction Between Masculine Norms and Alcohol Use

While alcohol use was mentioned throughout studies as a precursor to and risk factor for MFIPV perpetration (Pegram et al., 2018; Puri et al., 2010; Tlapek, 2016), further elaboration on the association between alcohol and violence was minimal. Instead, alcohol was discussed in terms of facilitating and amplifying the expression of masculine ideals and behaviors, a concept not explored by most quantitative studies. For example, Peralta et al. (2010) found alcohol use was aligned with strength and aggression, with many men identifying alcohol as a resource and solution for coping with feelings of masculine inadequacy. Pathways to this association were understood as beginning in childhood, within family and community settings, where drinking is viewed as “mature” and a masculine behavior. Findings by Tlakep (2016) further strengthen this interaction, with men referring to feeling “strong” when having alcohol and that they could do whatever they pleased when drinking. This is also highlighted by Wechsberg et al. (2013), where participants asserted that “men are supposed to drink,” portraying alcohol use as an accepted and expected behavior.

Despite these consistent findings, this interaction was not statistically supported in quantitative analyses, with Pegram et al. (2018) concluding there was no statistically significant pathway between heavy alcohol use (i.e., the quantity and frequency of use) and masculinity.

#### Contextual Variability in Masculinity Constructs

Differences in how masculinity is understood, the beliefs and behaviors it encompasses, vary across countries and cultural contexts, subsequently shaping dynamics and acceptability of IPV. Several studies, particularly those conducted in low-and middle-income countries such as Rwanda, Namibia and South Africa, framed masculinity in alignment with patriarchal norms, where male dominance is socially expected and actively reinforced (Duff et al., 2020; Tlapek, 2016; Wechsberg et al., 2013). For example, Puri et al. (2010) conceptualized masculinity within the context of arranged marriage and traditional cultural practices, where men are positioned as inherently superior, with violence an accepted and encouraged mechanism of asserting control when masculinity is perceived to be challenged.

Conversely, studies conducted in more Westernized cultures such as the USA, conceptualize masculinity through a psychological lens, focusing on internalized beliefs and attitudes rather than culturally embedded norms. For example, hostile masculinity as seen in Pegram et al. (2018), reflects attitudes that encompass distrust of women and support for sexualized male dominance rather than traditional cultural expectations. Similarly, qualitative findings by Peralta et al. (2010) show masculinity was closely tied to aggression, control and risk-taking behaviors (such as sexual dominance and violence) which were understood as expressions of masculine identity.

Despite differences in how masculinity is conceptualized, all studies acknowledge that these beliefs and practices ultimately reinforce male dominance over women in the context of MFIPV.

## Discussion

This systematic review and meta-analysis examined whether male alcohol use and masculinity intersect with MFIPV perpetration, while also assessing their individual associations with MFIPV. Across the six quantitative studies reviewed, the interaction of alcohol and masculinity with MFIPV was not significant. However, independently, heavier male alcohol use and stronger endorsement of masculine norms were significantly associated with MFIPV perpetration. Moreover, a narrative synthesis of five studies revealed consistent themes linking masculinity to dominance, with alcohol use as a mechanism that amplifies masculine beliefs and that highlights the contextual differences in how masculinity is constructed and enacted in relation to MFIPV. These findings are summarized in Table 2.

**Table 2.** Summary of Key Findings From This Review.

Both heavier male alcohol use and greater endorsement of masculine norms were independently associated with increased odds of MFIPV perpetration
Despite consistent qualitative evidence, the interaction between alcohol use and masculinity was not statistically significant in the meta-analysis
Very few studies examined how alcohol use and masculinity intersect with MFIPV perpetration, despite evidence establishing them as risk factors
Conceptualizations of masculinity varied across cultural contexts, shaping how masculinity was expressed and how it related to violence, from patriarchal norms in LMICs to hostile attitudes toward women in Western contexts
Narrative findings identified masculinity as a framework for dominance and control, often justified through authority, sexual entitlement and responses to perceived inadequacy
Alcohol was often described as amplifying masculine norms such as aggression and dominance, though this pathway is rarely examined quantitatively
A high level of methodological and conceptual heterogeneity was evident, partly due to variations in sample size, measurement scales and study region.

*Note.* MFIPV = male-to-female intimate partner violence.

### Independent Associations of Alcohol Use and Masculinity with MFIPV

Both male alcohol use and endorsement of masculine norms emerged as independently associated with increased odds of MFIPV perpetration, with alcohol demonstrating a stronger association than that observed for masculinity. However, differences in measurement and conceptualization of these two exposure variables warrant caution in directly comparing the magnitude of their effect sizes.

This review found a consistent association between male alcohol use and MFIPV perpetration across the included studies, reinforcing its role as a key risk factor. This aligns with prior reviews by Mootz et al. (2023) and Spencer et al. (2022) which identified significant associations between alcohol use and physical IPV perpetration among men. Specifically, Foran and O’Leary (2008) highlighted important distinctions between types of alcohol involvement, noting alcohol abuse or dependence is more strongly associated with IPV than frequency of use or binge drinking. Future research should further investigate these distinctions within diverse populations to better understand the alcohol and IPV association. Given the extensive body of research linking male alcohol use to increased risk of MFIPV (Graham et al., 2011; McKinney et al., 2010), the present findings are unsurprising. Studies show that when men drink, they tend to do so more heavily and for longer durations than women (Wilsnack et al., 2009), and that the odds of physical and sexual IPV increase with the number of drinks consumed (Shorey et al., 2014), further reinforcing the current results. Notably, a meta-analysis focused solely on sub-Saharan Africa found that male partner alcohol use was associated with a 141% increase in the odds of MFIPV perpetration (Tenkorang et al., 2021), substantially higher than the 39% increase identified in the present review. While the current study excluded some papers due to the requirement they also measure masculinity, this may have resulted in the omission of studies with stronger associations. Additionally, variation in cultural norms surrounding alcohol consumption likely contributed to differences in effect sizes. These factors highlight the need for future syntheses to consider both cultural variation and intersecting influences (e.g., masculinity and alcohol) across different populations to more accurately capture MFIPV risk.

Across the included studies, endorsement of masculine norms was also frequently linked to MFIPV perpetration. A clear pattern of positive associations were observed, with several previous reviews and meta-analytic examinations similarly identifying this relationship (Badenes-Sastre et al., 2024; Krivoshchekov et al., 2023; Ray & Parkhill, 2023; Spencer et al., 2022). While these reviews consistently reported significant positive associations, they varied in how masculinity was conceptualized, a theme reflected within the narrative synthesis. These definitions ranged from traditional masculinity (Krivoshchekov et al., 2023), traditional gender roles (Spencer et al., 2022), hostile masculinity (Ray & Parkhill, 2023) to favorable IPV attitudes (i.e., hostile/benevolent sexism and traditional gender roles; Badenes-Sastre et al., 2024). Notably, most of these review were conducted using studies from the USA or other high-income countries, making direct comparisons to the present review, which included a more culturally and geographically diverse sample, challenging. Although research globally has identified a consistent patterning of masculinities in relation to violence against women (Jewkes et al., 2020), the prevalence of MFIPV is highest in African, Eastern Mediterranean and South-East Asia regions (World Health Organization, 2021). Given qualitative syntheses highlighting strong links between masculinities and IPV in African contexts (Okeke-Ihejirika et al., 2019; Pelowich et al., 2024), there is a clear need for more research examining this association more broadly, as well as additional meta-analytic studies focused on harmful masculinities in regions with the highest MFIPV burden.

### Interaction Between Alcohol and Masculinity with MFIPV

Despite the independent associations of alcohol use and masculine norm endorsement with MFIPV perpetration, their interaction was not statistically significant. Given the small number of included studies (*n* = 6) and observations (*n* = 8) and evidence showing that meta-analyses with few studies typically lack the statistical power to detect significant associations (Turner et al., 2013), the current finding is unsurprising. Despite all included quantitative studies measuring alcohol use, masculinity and IPV, none examined the association between male alcohol use and masculine norm endorsement, a prominent theme identified among qualitative studies. Moreover, very few explored how these two factors intersect in relation to MFIPV perpetration. Given that gender-transformative research has identified this interaction in the context of HIV risk behaviors (Hatcher et al., 2022; Levtov et al., 2022), studies examining these concepts should be assessing the association of masculinity and alcohol use with MFIPV. This remains a critical gap in the literature and a necessary area for future research, particularly across diverse cultural and geographical contexts.

### Narrative Insights on Control, Alcohol, and Masculine Identity

In addition to the meta-analysis, the narrative synthesis provided important context, particularly regarding masculinity and alcohol. Links between masculinity and control align with research showing men often experience relationship power through exerting control over their partner (Harvey et al., 2002), with further evidence indicating greater hostile masculinity, characterizes by distrust of women and the use of sex as a form of dominance, is associated with MFIPV (Pegram et al., 2018). Qualitative findings also revealed connections between male alcohol use and perceptions of masculinity. However, despite the current review and earlier analyses (Willott & Lyons, 2012) identifying this relationship, only one included study assessed it quantitively, yielding a non-significant result. This discrepancy highlights the need for further quantitative research to explore whether this association holds statistical significance. Finally, contextual variability in the conceptualization of masculinity was evident both quantitatively and qualitatively. These differences align with R. Connell’s (2016) work, which emphasizes the fluidity and diversity of masculinities and the need to consider historical, cultural and global contexts in their definition. Accordingly, future research should continue to explore this variability to better understand how different dimensions of masculinity intersect with both alcohol use and MFIPV.

### Limitations

A major strength of this review is that it is the first to examine the intersection between male alcohol use and endorsement of masculine norms with MFIPV perpetration. Nevertheless, several limitations should be considered when interpreting findings. First, analysis of publication bias revealed the potential for small-study effects specific to masculinity outcomes, with Egger’s test identifying significant asymmetry (i.e., uneven distribution of ORs across studies), thus conclusions should be drawn cautiously. Second, as all included studies utilized a cross-sectional design, inferring causality or temporal order among alcohol use, masculinity and MFIPV is limited. Future research should prioritize longitudinal designs and consider multi-informant approaches, such as incorporating partner reports, to better capture how these factors influence each other over time and strengthen the reliability of findings. Third, studies were excluded if they grouped alcohol use with other drug use in the analysis, potentially omitting relevant data on the role of alcohol in MFIPV perpetration, particularly in contexts where polysubstance use is common. However, this highlights an avenue for future research to examine how engagement with both alcohol and other drugs intersects with both masculinity and violence. Fourth, variability in how masculinity was measured across studies may have influenced findings. While all instruments align with the conceptualization of masculinity for this review, prior research has shown that certain constructs (e.g., gender-equitable attitudes) can vary or show little variation within populations, and may not always align with related behaviors (Casey et al., 2024). Such variance or invariance in attitudes could affect the ability to detect associations and should be considered in future research. Finally, while gray literature such as dissertations and book chapters were included in selection criteria, other types of unpublished works were excluded, potentially reducing overall comprehensiveness of the review.

### Prevention and Policy Implications

The findings from this review highlight the need for integrated violence prevention strategies informed by both public health and gender-transformative approaches (see Table 3). The significant associations between both male alcohol use and masculine norm endorsement with MFIPV perpetration, alongside the identified relationship between alcohol and masculinity warrant interventions that address these risk factors collectively rather than in isolation (Peralta et al., 2010). This supports the WHO’s ecological framework for violence prevention (World Health Organization, n.d.), which emphasizes strategies that integrate individual, relationship, community and societal factors of violence against women.

**Table 3.** Implications for Policy, Prevention and Future Research.

Prevention
Develop integrated prevention strategies that address alcohol use and harmful masculinities together, not in isolation
Implement early school-based programs that challenge harmful gender norms and promote healthier masculinities
Target social meanings attached to male drinking, particularly where alcohol use reinforces dominance and aggression
Policy
Align prevention efforts with the WHO’s ecological framework, addressing risk across individual, relationship, community and societal levels
Ensure culturally responsive policies that consider diverse social norms surrounding alcohol and masculinity
Promote gender-transformative policies that shift systemic norms supporting male dominance and violence
Future research
Prioritize longitudinal and multi-informant research to explore causal pathways over time
Expand research across diverse cultural and geographical settings to help inform and develop context-specific interventions
Examine how engagement with both alcohol and other drugs intersects with both masculinity and violence
Greater analysis of the interaction between alcohol use and masculinity with MFIPV, particularly when data on these factors have already been collected for a study

*Note.* MFIPV = male-to-female intimate partner violence; WHO = World Health Organization.

While a statistically significant interaction between alcohol and masculinity was not observed, qualitative findings from this review suggest alcohol amplifies masculine expressions of dominance and aggression, traits consistently linked to violent intimate relationships. Given prior evidence from high- and low-income countries emphasizing the importance of integrating cultural differences and intersecting factors into intervention design and application (Heise, 2011; Jewkes et al., 2015), prevention programs should address how alcohol interacts with masculine identity in shaping violence, particularly the social meanings attributed to men’s drinking. Furthermore, implementation of early education programs focused on masculinity is crucial to support young boys in recognizing and challenging harmful gender norms while promoting healthier, non-violent models of masculinity.

## Conclusion

This systematic review and meta-analysis identified significant associations between both alcohol us and masculinity with MFIPV perpetration. Although the interaction of these two risk factors was not statistically significant, qualitative studies described a clear relationship between alcohol and masculinity. These findings highlight the intersectional nature of alcohol, masculinity and violence, while also pointing to a lack of studies that analyse these relationships, despite having collected the relevant data to do so. The findings further underscore the variability in how masculinity is conceptualized, emphasizing the importance of examining how masculinities manifest and intersect with alcohol and violence across diverse cultural and global contexts. Future research should aim to deepen understanding of the associations between alcohol use, masculine norm endorsement and MFIPV perpetration to better inform the development of integrated interventions and policies that address these interconnected factors.

## Supplemental Material

sj-docx-1-tva-10.1177_15248380251383937 – Supplemental material for Understanding the Intersection Between Men’s Alcohol Use, Masculinity and Perpetration of Intimate Partner Violence: A Systematic Review and Meta-Analysis Supplemental material, sj-docx-1-tva-10.1177_15248380251383937 for Understanding the Intersection Between Men’s Alcohol Use, Masculinity and Perpetration of Intimate Partner Violence: A Systematic Review and Meta-Analysis by Bree Willoughby, Koen Smit, Ingrid M. Wilson, Gail Gilchrist, Cassandra Hopkins, Amany Tanyos, Miro Saunders, Gedefaw Diress Alen and Anne-Marie Laslett in Trauma, Violence, & Abuse
