# Supplementary material for: Understanding the Intersection Between Men’s Alcohol Use, Masculinity and Perpetration of Intimate Partner Violence: A Systematic Review and Meta-Analysis
Source: Trauma Violence Abuse. 2025 Nov 12;27(5):2579–96. doi: 10.1177/15248380251383937 (PMC13736298; doi:10.1177/15248380251383937)
Supplement: Supplementary file 1 [file sj-docx-1-tva-10.1177_15248380251383937.docx]

**Supplementary Table 1.** Database search terms

| **Search ID#** | **Search Terms** | **Search Notes** |
| --- | --- | --- |
| S1 | Alcohol Drinking/ | Subject heading (MeSH) |
| S2 | Binge Drinking/ | Subject heading (MeSH) |
| S3 | "alcohol use" OR "alcohol abuse" OR "alcoholism" OR "binge drinking" OR OR "alcohol* drink*" OR "alcohol misuse" OR "harmful alcohol use" OR "alcohol use disorder" OR "alcohol* intoxicat*" OR intoxicat* OR "risky alcohol use" OR "hazardous drink*" OR "alcohol addiction" OR "alcohol dependen*" OR "problem* drink*" OR "risky drink*" OR "high risk drink*" OR "alcohol consumption" OR "alcohol intake*" OR drunkenness* OR "alcoholic" | Keyword |
| S4 | S1 OR S2 OR S3 | Combination OR |
| S5 | Masculinity/ | Subject heading (MeSH) |
| S6 | "masculin*" OR "manhood" OR "manl*" OR "macho" "male dominance" OR “masculine norms” OR “masculine ideology” OR “hyper-masculin*” OR “men” OR “masculine ideals” OR “traditional masculinity” OR “hegemonic masculinity” | Keyword |
| S7 | “normative beliefs” OR “sexism” OR “sexist” OR “misogyny” OR “traditional gender roles” OR “gender stereotypes” OR “gender inequality” OR “sex roles” OR “gender norms” OR “Gender Role Strain” OR “Gender-Equitable Men scale” OR “GEM scale” OR “Conformity to Masculine Norms Inventory” OR “Gender Roles Attitude Scale” OR “Attitudes Toward Women Scale” OR “attitudes toward women” OR “Sex-Role Egalitarianism Scale” OR “Modern Sexism” OR “Ambivalent Sexism Inventory” OR “BEM Sex-Role Inventory” | Keyword |
| S8 | S5 OR S6 OR S7 | Combination OR |
| S9 | Intimate Partner Violence/ | Subject heading (MeSH) |
| S10 | Domestic Violence/ | Subject heading (MeSH) |
| S11 | "intimate partner violence" OR "IPV" OR "domestic violence" OR "domestic abuse" OR "spous* abuse*" OR "violence against wom?n" OR "partner abuse" OR "abused wom?n" OR "wife beating" OR "partner aggression" OR "dating violence" OR "sexual* abus$*" OR "sexual* aggress*" OR "sexual* assault*" OR "rape" OR "fear" OR "physical* violen*" OR "physical* assault*" OR "physical* abus*" OR "physical* aggress*" OR "emotional* abus*" OR "psychological* abus*" OR "psychological* aggress*" OR "coercive control" OR "control* behavio?r*” OR "intimate partner homicide" OR "domestic homicide" OR “hostility toward women” | Keyword |
| S12 | S9 OR S10 OR S11 | Combination OR |
| S13 | S4 AND S8 AND S12 | Combination AND |

**Supplementary Table 2.** Assessment of risk of bias for all included studies using the Mixed-methods Appraisal Tool

|  | **Quantitative non-randomised** | | | | | | |
| --- | --- | --- | --- | --- | --- | --- | --- |
| **Author & Year** | Are there clear research questions? | Do the collected data allow to address the RQ’s? | Are the participants representative of the target population? | Are the measurements appropriate regarding both the outcome and intervention (or exposure)? | Are there complete outcome data? | Are the confounders accounted for in the design and analysis? | During the study period, is the intervention administered (or exposure occurred) as intended? |
| Abrahams et al. (2006) | Yes | Yes | Yes | Yes | Yes | Yes | Yes |
| Cantor et al. (2021) | Yes | Yes | No | Yes | Yes | No | Yes |
| Dasgupta et al. (2018) | Yes | Yes | Yes | Yes | Yes | Yes | Yes |
| Gilchrist et al. (2017) | Yes | Yes | Yes | Yes | Yes | Yes | Yes |
| Gottert et al. (2018) | Yes | Yes | Yes | Yes | Yes | Yes | Yes |
| Laslett et al. (2021) | Yes | Yes | Yes | Yes | Yes | Yes | Yes |
| Lisco et al. (2012) | Yes | Yes | Yes | Yes | Yes | No | Yes |
| Lisco et al. (2015) | Yes | Yes | Yes | Yes | Yes | Yes | Yes |
| Lynch & Renzetti (2020) | Yes | Yes | Yes | Yes | Yes | Yes | Yes |
| Machisa et al. (2016 | Yes | Yes | Yes | Yes | Yes | Yes | Yes |
| Machisa & Shamu (2018) | Yes | Yes | Yes | Yes | Yes | Yes | Yes |
| Miedema et al (2022) | Yes | Yes | Yes | Yes | Yes | Yes | Yes |
| Pegram et al. (2018) | Yes | Yes | Yes | Yes | Yes | Yes | Yes |
| Renzetti et al. (2018) | Yes | Yes | Yes | Yes | Yes | Yes | Yes |
| Warkentin (2010) | Yes | Yes | Yes | Yes | Yes | Yes | Yes |
|  | **Qualitative studies** | | | | | | |
|  | Are there clear research questions? | Do the collected data allow to address the RQ’s? | Is the qualitative approach appropriate to answer the RQ? | Are the data collection methods adequate to address the RQ? | Are the findings adequately derived from the data? | Is the interpretation of results sufficiently substantiated by the data? | Is there coherence between data sources, collection, analysis and interpretation? |
| Duff et al. (2020) | Yes | Yes | Yes | Yes | Yes | Yes | Yes |
| Puri et al. (2010) | Yes | Yes | Yes | Yes | Yes | Yes | Yes |
| Wechsberg et al. (2013) | Yes | Yes | Yes | Yes | Yes | Yes | Yes |
|  | **Mixed-methods studies** | | | | | | |
|  | Are there clear research questions? | Do the collected data allow to address the RQ’s? | Is there an adequate rationale for using a mixed-methods to address the RQ? | Are the different components of the study effectively integrated to answer the RQ? | Are the outputs of the integration of qualitative and quantitative components adequately interpreted? | Are divergences and inconsistencies between quantitative and qualitative results addressed adequately? | Do the different components adhere to the quality criteria of each tradition of the method involved? |
| Peralta et al. (2010) | Yes | Yes | No | No | No | Can’t tell | No |
| Tlapek (2016) | Yes | Yes | Yes | Yes | Yes | Yes | Yes |
